# Transcriptional responses of *Trichodesmium* to natural inverse gradients of Fe and P availability

**DOI:** 10.1038/s41396-021-01151-1

**Published:** 2021-11-24

**Authors:** E. Cerdan-Garcia, A. Baylay, D. Polyviou, E. M. S. Woodward, L. Wrightson, C. Mahaffey, M. C. Lohan, C. M. Moore, T. S. Bibby, J. C. Robidart

**Affiliations:** 1 Ocean and Earth Science, University of Southampton, Southampton, SO14 3ZH UK; 2 National Oceanography Centre, Southampton, SO14 3ZH UK; 3 Plymouth Marine Laboratory, Plymouth, PL1 3DH UK; 4 Earth, Ocean and Ecological Sciences, University of Liverpool, Liverpool, L69 3BX UK

**Keywords:** Biogeochemistry, Transcriptomics, Microbial ecology

## Abstract

The filamentous diazotrophic cyanobacterium *Trichodesmium* is responsible for a significant fraction of marine di-nitrogen (N_2_) fixation. Growth and distribution of *Trichodesmium* and other diazotrophs in the vast oligotrophic subtropical gyres is influenced by iron (Fe) and phosphorus (P) availability, while reciprocally influencing the biogeochemistry of these nutrients. Here we use observations across natural inverse gradients in Fe and P in the North Atlantic subtropical gyre (NASG) to demonstrate how *Trichodesmium* acclimates in situ to resource availability. Transcriptomic analysis identified progressive upregulation of known iron-stress biomarker genes with decreasing Fe availability, and progressive upregulation of genes involved in the acquisition of diverse P sources with decreasing P availability, while genes involved in N_2_ fixation were upregulated at the intersection under moderate Fe and P availability. Enhanced N_2_ fixation within the Fe and P co-stressed transition region was also associated with a distinct, consistent metabolic profile, including the expression of alternative photosynthetic pathways that potentially facilitate ATP generation required for N_2_ fixation with reduced net oxygen production. The observed response of *Trichodesmium* to availability of both Fe and P supports suggestions that these biogeochemically significant organisms employ unique molecular, and thus physiological responses as adaptations to specifically exploit the Fe and P co-limited niche they construct.

## Introduction

In low nitrogen (N) marine environments, diazotrophs, organisms capable of fixing atmospheric di-N into biologically available ammonia (NH_3_) [1, 2] contribute to a significant fraction of new N input in the oceans. The colonial cyanobacterium *Trichodesmium* is one of the main contributors to the fixed N budget of the world’s oceans [3, 4] accounting for up to 50% of total upper ocean N_2_ fixation in some areas [5] with an estimated annual input of 60–80 Tg N [6]. *Trichodesmium* can be the dominant diazotroph in regions such as the North Atlantic subtropical gyre (NASG), fuelling biological productivity and driving biogeochemical cycles[5–7].

*Trichodesmium*’s distribution and N_2_ fixation activity is restricted by the availability of nutrients such as iron (Fe) and phosphorus (P) [7–9]. This is distinctly evident across latitudinal gradients in the Atlantic, where low dust Fe flux south of the inter-tropical convergence zone (ITCZ) in the South Atlantic subtropical gyre limits the activity of *Trichodesmium* and other N_2_ fixers, resulting in relatively high residual P concentrations [9, 10]. In contrast, increased dust flux north of the ITCZ provides sufficient Fe for *Trichodesmium* to flourish and thus drawdown surface P to nanomolar concentrations [7, 11–13]. Such boundaries between high-Fe/low-P and low-Fe/high-P regions thus represent constructed niches which appear widespread in low latitude oligotrophic oceans [9, 11, 14]. Moreover, evidence for Fe and P co-limitation [15] and observed physiological responses of *Trichodesmium* under these conditions have led to suggestions that the organism may be specifically adapted to nutrient co-stress [16–19].

Culture and field studies of *Trichodesmium* using gene expression analysis [20], proteomic profiling [19, 21] or both [18, 22] have provided insights into *Trichodesmium*’s ecophysiology, including the identification of biomarker indicators of nutrient stress [17, 20, 23]. Given the importance of Fe and P availability, *Trichodesmium* has evolved several acclimation strategies to cope when these key resources are restricted. This is particularly true for Fe, given its absolute requirement in both the catalysis of N_2_ fixation and photosynthesis [24, 25]. *Trichodesmium* does not temporally separate photosynthesis from N_2_ fixation and as such cannot ‘share’ Fe between these processes, potentially increasing Fe requirements relative to other diazotrophic groups such as *Crocosphaera* [16, 21]. In response to Fe scarcity, *Trichodesmium* reduces demand through reduction of high Fe content enzymes including photosystems I and II (PSI and PSII) and nitrogenase [21, 24] and replacement of Fe-containing enzymes with non-Fe dependent isozymes such as ferredoxin with flavodoxin [26] and cytochrome c_533_ with the Cu-dependent plastocyanin [27]. Moreover, *Trichodesmium* potentially increases Fe supply through upregulation of Fe transporters (*IdiA*/FutA) [28, 29]. Simultaneously performing photosynthesis and N_2_ fixation generates a further challenge for *Trichodesmium* in generating the ATP and reductant required for N_2_ fixation while minimising net oxygen production to protect nitrogenase from oxygen inhibition [30]. While regulation over the light cycle [31] and reversible cellular differentiation into proposed diazocytes [4, 32] can help achieve this, the molecular regulation that enables simultaneous aerobic and anaerobic chemistry is not fully characterised.

*Trichodesmium* can access P in various forms 33]. In addition to acquisition of dissolved inorganic phosphate (DIP) (including phosphate and phosphite), *Trichodesmium* can utilise a broad range of dissolved organic P (DOP) compounds when DIP is low, which is used as an indication of P stress state. These compounds include phosphomonoesters and phosphonates [34], which can be found at higher concentrations in some oligotrophic regions[35]. A range of P-stress genes and protein biomarkers involved in P-acquisition have thus been characterised, such as the high-affinity inorganic P transporters *pstS* and *sphX*, the putative alkaline phosphatases (APs) *phoA* and *phoX* [22, 33], the phosphonate-related *phnCDEEGHIJKLM* [34], and the phosphite utilisation *ptxABCD* genes [36].

Here, *Trichodesmium’*s transcriptomic response and relative abundance within the diazotrophic community was investigated across naturally opposed gradients in Fe and P concentrations. A reduced capability for N_2_ fixation is observed at both ends of the transect, where either Fe or P are at their lowest concentrations. Elevated N_2_ fixation rates in the transition region between the areas of lowest P and lowest Fe availability coincided with a consistent gene expression profile, suggesting an increased capacity to fix N_2_ and produce cobalamin (B_12_ vitamin) at intermediate nutrient concentrations. In addition, the enhanced N_2_ fixation capacity may be supported by alternative ATP generation pathways that are both Fe-efficient and do not generate net O_2_. The observed functional acclimatisation dependent on Fe and P availability may be key contributing factor in the ecological success of *Trichodesmium* across oceanic gyres [14].

## Materials and methods

### Hydrography and environmental data

**Fig. 1:**
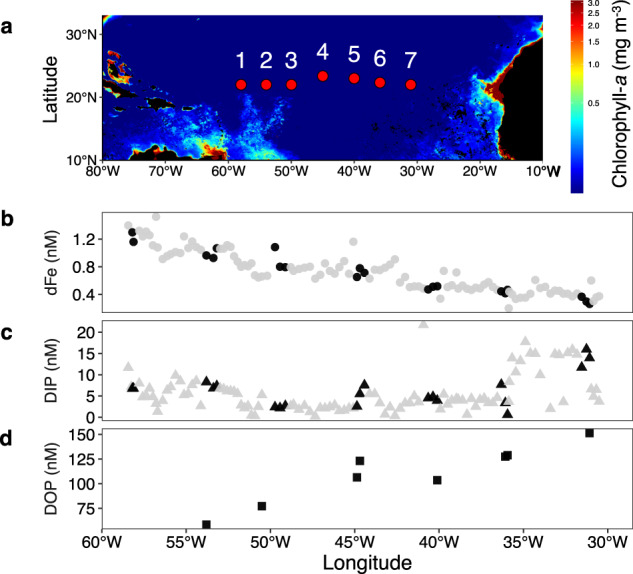
Transect and surface nutrient profiles. **a** Sampling stations 1–7 for the RRS James Cook-JC150 (GEOTRACES GApr08) cruise across the subtropical North Atlantic Ocean (June–August 2017 at ~22 ^o^N) from Guadeloupe (French Caribbean) to Tenerife (Canary Islands). Background colour shows the satellite-derived chlorophyll-*a* concentration (mg/m^3^) from July 2017 [100]. Surface nutrient concentrations (nM) for (**b**) dFe, (**c**) DIP and (**d**) DOP (in grey) including measurements from the seven sampling stations (in black). dFe is a sub-set of data from Kunde et al. [38].

Sampling was carried out on board the RRS James Cook JC150 (GEOTRACES GApr08) in the subtropical North Atlantic on a transect from Guadeloupe (French Caribbean) to Tenerife (Canary Islands) (26th June to 12th August 2017) (Fig. 1a). Surface (2–3 m) underway water sampling for nutrient and Fe analysis was conducted using a Teflon diaphragm pump (Almatec A-15) connected to a “Towed-Fish”, which pumped seawater into a class-1000 clean-air laboratory. Samples for dissolved Fe (dFe) were filtered in-line (<0.2 μm; Sartobran) and measured using flow injection with chemiluminescence detection [37] as reported in Kunde et al. [38]. The detection limits were 0.03 ± 0.02 nM (*n* = 59). Nanomolar measurements of DIP were made using colorimetric method with segmented flow analysis coupled to a 2 m liquid waveguide as the analytical flow cell to improve detection limits [39]. DIP detection limit was 1 nM (3 × the Milli-Q water baseline, determined over the course of the research cruise). Certified reference materials from KANSO Technos (Japan) were measured for quality control. Total dissolved phosphorus (TDP) was determined using the high temperature acid persulfate technique as in Davis et al. [40]. DOP was taken as the difference between TDP and DIP (DOP = TDP-DIP, where DOP detection limit = 40 nM; TDP-DIP, thus 2 × DL of DIP (20 nM)) [41]. In situ N_2_ fixation rates were measured using the modified bubble injection method described by Klawonn et al. [42]. Specifically, 8 mL of 98% ^15^N_2_ gas (Cambridge Isotope Laboratories, NLM-363-0, lot No: I-21065/AR0664758) was added to each 4.2 L incubation bottle through the septa. Bottles were strapped to a cable drum, rolled across the deck (~10 rpm) for 15 min. 20 mL of seawater was removed (4200 − 20 mL = 4180 mL, 0.47% volume) and filtered seawater was added to displace the volume removed, ensuring there was no bubble remaining in the bottle before the incubation (Supplementary information). Samples were analysed on board using a Hiden Membrane Inlet Mass Spectrometer (Supplementary information).

### Sample collection

*Trichodesmium* colonies were collected at seven stations along the transect (Fig. 1a) using plankton nets (200 μm mesh, 50 cm diameter) (Duncan & Associates, UK) deployed at 15 meters for 15 min. Net cod-ends were emptied into an acid-cleaned bucket and transferred to a laminar flow hood inside a trace-metal clean bubble laboratory. Samples of 50 colonies (in triplicate) were randomly hand-picked using plastic Pasteur pipettes and individually rinsed through three steps (0.2 μm filtered “Towed-Fish” seawater) before allocation into 150 mL bottles (previously HCl rinsed and filled with filtered <0.2 μm “Towed-Fish” seawater). Bottles were filtered onto 0.2 μm Supor filters (Sigma-Aldrich, St. Louis, USA), flash frozen in liquid N_2_ and stored at −80 °C. Samples were collected at the same time-point to remove the influence of any diel transcriptional variability. For this study we prioritised daybreak (5–6am local time) to measure transcription at peak nitrogenase, N metabolism, P metabolism and photosystem transcription according to Frischkorn et al. [43]. It is possible that this choice relegated transcripts from other gene families (troughs in diel transcription) to below the limit of detection. These include transcripts coding for amino acid metabolism, ATP synthesis, ribosomes and some cofactors and vitamins. In this manuscript we restrict our analyses to transcripts that are differentially expressed, and present in all samples across the transect.

### DNA/RNA extractions

RNA extractions used a modification of the AllPrep DNA/RNA mini kit (Qiagen, Manchester, UK), DNase digestion (Qiagen DNase kit) and clean-up (ZYMO RNA Clean and Concentrator) as described in Tang et al. [44]. RNA concentrations were measured using Qubit (Qiagen) and extracts were stored at −80 °C in 15 μL aliquots for metatranscriptomic sequencing.

### Quantitative polymerase chain reaction (qPCR)

Targeted quantitative Polymerase Chain Reaction (qPCR) approaches were used to identify and quantify the *Trichodesmium* and UCYN-A *nifH* genes along the cruise transect (Supplementary Fig. 1). Gene copies were quantified relative to standard curves obtained from the amplification of linearised plasmids with the targeted gene inserts. Standard gene dilution series (10^7^−10^0^ copies L^−1^) were made with nuclease-free H_2_O (Ambion, CA, USA) for each reaction and negative controls contained nuclease-free H_2_O in place of DNA template (Methods described in Supplementary information).

### DNA/RNA sequencing

#### nifH amplicon sequencing

Nested degenerate polymerase chain reaction (PCR) was used to amplify the *nifH* genes using degenerate primers [45] and second round PCR primers *nifH*1 and *nifH*2 [46] with Illumina tag sequences as described elsewhere [44, 47]. in PCR reactions were cleaned and concentrated using the MinElute kit (Qiagen) and Nextera XT indexed PCR was used to add i5/i7 sequencing adaptors (Part 15044223 Rev. B, Illumina). Equimolar concentrations of *nifH* amplicons were pooled and sequenced on the MiSeq (Illumina) at the University of Southampton’s Environmental Sequencing Facility.

#### Whole transcriptome sequencing

Depletion of rRNA from total RNA was performed using the Ribo-Zero Bacteria Magnetic Kit (Illumina). RNA-Seq libraries were prepared in triplicate for each sample for MiSeq (Illumina) sequencing. The library was prepared using the ScriptSeq v2 RNA-Seq Library Preparation Kit Illumina (originally EpiCentre), implementing a tagging oligo method [48] to determine which strand the transcript was from.

### Metagenome sequencing and assembly

Metagenome assembly came from a set of 56 single *Trichodesmium* colonies collected from 21st to 27th July 2016 aboard the RV Atlantic Explorer in the Sargasso Sea (NCBI BioProject ID PRJNA721834). Colonies were picked, filtered and frozen as described above (see section *Sample collection*). DNA was extracted with the DNeasy Plant mini kit (Qiagen) according to the manufacturer’s protocol. DNA extracts from five T. *thiebautii* colonies were chosen for whole genome sequencing (based on DNA concentration). Library preparation used KAPA HyperPlus DNA library prep kit following manufacturer’s instructions. Libraries were pooled and 2 × 300 bp paired-end sequencing was carried out on a MiSeq (Illumina) using a MiSeq v3 Reagent Kit (Illumina). Reads were trimmed using Cutadapt version 2.3 [49]. Initial assemblies were carried out using SPADES version 3.13.1 [50] in metagenome mode. Resulting contigs were binned using MaxBin [51], followed by CheckM [52]. All five samples yielded a distinct cyanobacterial bin estimated to be >97% complete based on marker gene analysis. To identify *Trichodesmium-*only reads, tetranucleotide frequency profiles for all metagenome contigs of length >5 kb were compared to those of randomly simulated fragments (500 fragments, mean length 10 kb, standard deviation 1 kb) from the IMS101 genome sequence (Supplementary Fig. 2). Original read sets were filtered to extract *Trichodesmium* reads by alignment to the binned contigs using Bowtie2 [53] and reassembled using SPADES. We picked assembly “sct-8t3” as a reference genome as it was the most contiguous.

Putative coding sequences were identified on the metagenome contigs using Prodigal version 2.6.3 [54] with default settings. The resulting 6669 predicted protein sequences were clustered into 4044 orthologous groups (OGs) using MCL version 14-137 [55] (inflation parameter 1.4) [43]. OGs were characterised by comparison against IMS101 protein sequences, and the SEED [56] and UniRef90 databases using DIAMOND [57]. Consensus annotations for each OG were chosen by taking the most abundant SEED and UniRef90 annotations across the proteins making up each OG, as has been previously described [23, 43]. Where different annotations were equally abundant within an OG, the hit with the highest bit score was chosen.

### Bioinformatics

#### Amplicon sequencing

*nifH* relative amplicon abundance was determined using QIIME [58] as in Tang et al. [44] Raw sequences were merged and quality filtered (quality score >20 and read length >200 bp) using USEARCH [59]. Following a chimera removal step, sequences were clustered into operational taxonomic units (OTUs) at 97% sequence similarity using UPARSE [60]. The *nifH* sequences obtained were assigned taxonomies using the BLAST resource on NCBI.

#### Metatranscriptome dataset

Raw sequence quality was assessed (FastQC version 11.9), before pre-processing, annotation and analysis using the SAMSA2 metatranscriptomic analysis pipeline [61]. Low-quality and short sequences and adaptors were trimmed with Trimmomatic v0.3 [62], followed by removal of RNA ribosomal sequences with SortMeRNA [63]. Paired-end reads were merged with PEAR v0.9.10 [64]. Cleaned sequences were annotated by DIAMOND sequence aligner (version 0.8.3) mapped against NCBI’s most recent RefSeq non-redundant protein database (created January 2019) [57, 65], and against SEED [56] for hierarchical clustering of functional annotations. Mapping rates to *Trichodesmium* averaged 36% across samples, similar to a previously published metatranscriptome (37%; [23, 43]). Reads were also mapped to contigs from the custom T. *thiebautii* metagenome from the Sargasso Sea (described above) using Hisat2 version 2.2.12 [66] in strand-specific mode. Reads mapping to predicted coding sequences were counted using featureCounts [67]. Using the Sargasso Sea reference, we obtained higher mapping rates (41.92%; Supplementary Table 1), which could be due to the inclusion of genes from other *Trichodesmium* species in the field in addition to T. *erythraeum* IMS101, as explained in Rouco et al. [23] The broad data interpretations were similar when using the more limited IMS101 mapping. Therefore, the custom metagenome was used for subsequent analysis.

### Differential expression and statistical analyses

Differential gene expression analysis was carried out using the R (4.0.3) package DESeq2 (1.28.1) [20, 68, 69]. DESeq2 only considers genes that fit into a binomial distribution and are differentially expressed between stations. The threshold to include transcripts into the analysis depends on sample size and the number of parameters to be estimated. OG relative abundances were estimated from the package negative binomial generalised linear model fit for each OG. Significance was calculated through pairwise comparisons between stations by a likelihood ratio test with an adjusted *p* value (<0.1) for multiple test correction, similar to Walworth et al. [17] Differential expression was calculated relative to the Rota housekeeping OG [23, 33].

Normal distribution of data was based on the total library size using the median ratio method implemented in DESeq2 [68]. Pearson parametric tests (*p* value level of significance 0.05) were used for the correlations between the normalised gene expression across all samples (*n* = 17) and measured environmental conditions.

## Results and discussion

### Environmental and hydrographic data

Seven stations were sampled along a west-east transect across the NASG at ~22 °N (Fig. 1a). A west to east decrease of surface total dissolved iron (dFe) concentrations was observed, decreasing from 1.23 to 0.26 nM (Fig. 1b), consistent with other oligotrophic North Atlantic studies [70, 71] and reflecting the influence of advection in the west [38]. Opposing the dFe gradient, an increasing eastward trend was observed for DOP and total dissolved surface P (TDP), while DIP was slightly elevated at the easternmost stations (6 and 7) (Fig. 1c, d). The low concentrations of surface DIP (0.6–15 nM) and DOP (72–150 nM) were consistent with conditions typically observed in the North Atlantic [7, 12, 72]. The dFe:TDP ratio thus decreased from west (12.3 ± 2.6 mmol:mol) to east (2.0 ± 0.2 mmol:mol) (Supplementary Fig. 3), stations 3, 4 and 5 (mid-transect) being located within the transition zone between the higher relative concentrations of dFe and TDP. Notably, there was no zonal trend in DIN (Supplementary Table 2).

### Diazotroph diversity and *Trichodesmium* abundance

**Fig. 2:**
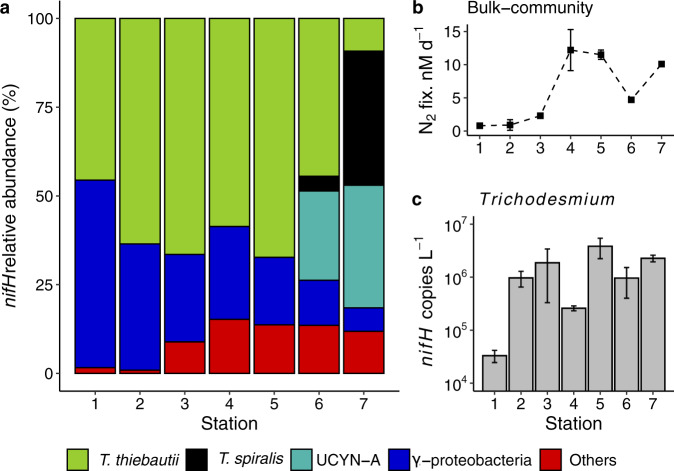
Diazotroph abundances and measured bulk N_2_ fixation rates. **a** Relative abundance of the *nifH* amplicon for *Trichodesmium* sp. (*T. thiebautii, T. spiralis)*, UCYN-A, γ-Proteobacterium 24774A11 and other non-identified *nifH* sequences. **b** In situ bulk measured N_2_ fixation rates (nM per day). **c** *Trichodesmium nifH* gene abundance (genes L^−1^) quantified by qPCR.

Diazotroph community composition was revealed by *nifH* gene (which encodes a core subunit of the nitrogenase enzyme responsible for N_2_ fixation) ([73] Fig. 2a) sequencing. In addition to *Trichodesmium*, a number of microorganisms actively fix atmospheric N_2_ in oligotrophic systems [74]. Total *nifH* sequence diversity increased from west to east, and *Trichodesmium* spp. sequences dominated throughout, averaging ~57% of total *nifH* amplicon sequences (ranging between 46 and 67%), similar to other studies from the region [75]. *T. thiebautii* was identified as the dominant *Trichodesmium* species at stations 1–6, whereas *T. spiralis* was the dominant species at the easternmost station 7, where dFe:TDP was lowest. Despite their morphological differences, both *Trichodesmium* species belong to the same phylogenetic clade (Clade I) showing different biogeography [76]. The widespread and abundant gamma-proteobacteria group γ-24774A11 [77, 78] was a significant part of the diazotroph community, averaging 25% of total *nifH* DNA sequences, gradually decreasing in relative abundance from west to east (57–7%). Lastly, the unicellular UCYN-A was only detected at the easternmost stations (st. 6,7) representing 25% and 34% of total *nifH* DNA sequences, respectively, potentially suggesting UCYN-A is competitive under lower dFe:TDP, and gamma-proteobacteria under higher dFe:TDP. A strong positive relationship between UCYN-A and phosphate concentration has been observed previously [79] including in the North Atlantic [44], though this relationship doesn’t hold for all regions [80]. While the gamma-proteobacteria have been found to be more abundant at higher temperatures [81, 82], their relationship with nutrients is not as clear. In situ community N_2_ fixation rates ranged from 0.8 to 18.2 nM d^−1^ (Fig. 2b), with the highest rates at the mid-transect stations 4 and 5, suggesting active N_2_ fixation may potentially be restricted by dFe in the east, and P in the west over this transect. This contrasts with a previous west-to-east transect at 10 °N which measured highest rates to the east of this transect (16–25 °W), corresponding to higher Fe concentrations nearer the coast [83].

In addition to dominating the *nifH* amplicon sequence data, *Trichodesmium* spp. *nifH* genes were detectable by qPCR across the transect, ranging from 10^4^ to 10^6^ copies L^−1^ (Fig. 2c) with a peak (3.8 × 10^6^ copies L^−1^) at station 5 in the middle of the transect, corresponding with one of the highest N_2_ fixation rates (11.5 nM d^−1^) (Fig. 2c). *Trichodesmium nifH* abundances were also high at stations 4 and 7 (2.6 × 10^5^ copies L^−1^ and 2.3 × 10^6^, respectively), corresponding with high rates of N_2_ fixation.

### Functional characterisation of *Trichodesmium*

**Fig. 3:**
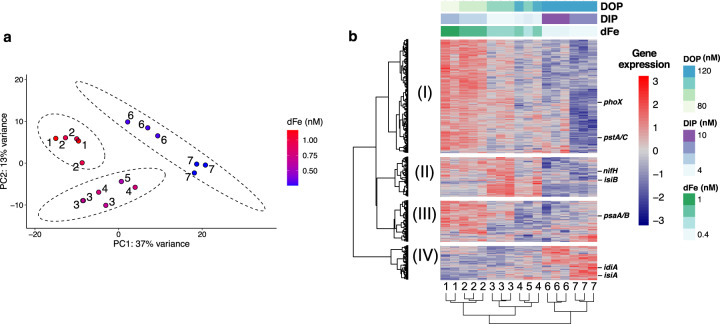
*Trichodesmium* metatranscriptome. **a** Principal component analysis (PCA) of all the 4044 *Trichodesmium* OGs across stations from all 17 samples calculated from the *variance stabilising transformation* (VST) using DESEq2 [68]. Colour gradient reflects measured surface dFe concentrations (0.3–1 nM). Numbers represent the sample stations. **b** Transcript expression patterns of the 1057 most differentially expressed OGs (DEOGs) across stations. Columns are individual samples clustered based on basis of Euclidean distance annotated with station number. Colour scale indicates high (red) to low (blue) DESEq2 VST OG normalised abundance scaled per row. Four clusters of expression patterns (labelled I–IV) were obtained from k-mean optimal clustering by Euclidean distance. Corresponding nutrient concentrations are shown above heatmap (nM).

Metatranscriptomic analysis was performed on hand-picked *Trichodesmium* colonies from stations 1–7 in triplicate. Sequencing was successful on 17 of the 21 samples collected, with an average of 1,208,214 mRNA reads (ranging from 471,088 to 2,260,415 reads) per sample (Supplementary Table 1). OGs of *Trichodesmium* transcripts were examined using a Principal Component Analysis (PCA) on hierarchical associations from Euclidean distances based on expression variability among samples (*n* = 17). Stations clustered into three distinct groups (Fig. 3a), which presented strong reproducibility among biological replicates from each station and coincided with the geographic distribution of stations as well as their characteristic nutrient environments (Fig. 1, Supplementary Fig. 3). As such, three distinct metatranscriptome regions were defined.west: stations 1–2 where dFe was highest, DOP lowest and hence dFe:TDP was higher; mid-transect: stations 3–5 from the middle of transect where N_2_ fixation rates were highest; and eeast: stations 6–7 where dFe was lowest, DOP highest and hence dFe:TDP lowest. The PCA analysis of the global transcriptome clearly demonstrates that *Trichodesmium* spp. alter gene expression profiles across the observed natural gradients in Fe and P availability.

The transcript analysis across stations revealed significant differential expression for 26.1% (1057 of 4044) of *Trichodesmium* OGs (adjusted *p* value < 0.1), hereafter termed DEOGs. Spatial DEOG patterns reflected the geographic clusters: (I) upregulated in the west (*n* = 526); (II) up- (*n* = 183) or (III) downregulated in the centre of the gyre (*n* = 190); and (IV) upregulated in the east (*n* = 158) (Fig. 3b; Supplementary Fig. 4). OGs were most differentially expressed between stations 2 and 7 at opposite ends of the gyre (935 differentially expressed OGs in total, representing 23.1% of the total OGs) (Supplementary Table 3). Remarkably, just 3 OGs (0.07%) were differentially expressed between stations 1 and 2 in the west. While the west cluster was distinct from the cluster in the central gyre (stations 3, 4, and 5), expression was fairly conserved in the centre cluster, with a maximum variation of 31 OGs, or 0.77% of the total expressed OGs between stations. The east cluster (stations 6 and 7) showed the greatest within-group diversity, explained by 358 DEOGs (8.8% of the total OGs) between the two stations, of which 61% (219 OGs) had unknown function (not shown). This diversity may reflect species-specific differences in gene expression as*Trichodesmium spiralis* became the dominant *nifH* sequence at station 7 (Fig. 2a), while *Trichodesmium thiebautii nifH* was dominant at all other stations.

To confirm that our conclusions were not affected by our choice of metagenomic reference sequence, this analysis was repeated using the binned *Trichodesmium* metagenomic contigs described in previous work [23, 43]. Frischkorn via personal communication). Mapping rates to this reference were similar, but slightly lower than to the Sargasso Sea reference (37% reads assigned to features vs 42%). Following OG clustering and quantification, a similar transcriptomic profile across the transect was obtained (Supplementary Fig. 5).

To further investigate the functions behind these transcriptional differences across regions, DEOGs were clustered into gene functional categories using SEED [56] (Supplementary Fig. 6). Photosynthesis, carbohydrates and N metabolism represented the categories with highest relative OG abundances across stations, in accordance with the main metabolic activities and biogeochemical roles of *Trichodesmium*. From a broad functional perspective, the high dFe:TDP region in the west caused upregulation of P metabolism, cell division and fatty acids. In addition, the high N_2_ fixation mid-transect region had upregulation of N metabolism, and downregulation of photosynthesis. Lastly, the low dFe:TDP eastern region had higher membrane transport and Fe metabolism transcription (Supplementary Fig. 6a). However, these functional category levels included a variety of OGs with cosmopolitan distribution throughout the dataset (Supplementary Fig. 6b). Specific photosynthetic OGs identified were differentially regulated throughout transect while subcategories of N metabolism such as N_2_ fixation were separate from ammonia or nitrite assimilation (Supplementary Fig. 6b).

### Nutrient stress biomarkers

**Fig. 4:**
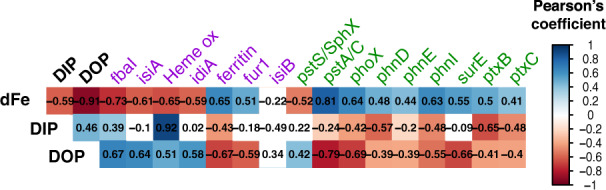
*Trichodesmium* nutrient stress. Correlation matrix of normalised counts from selected gene OG associated with Fe (blue) and P (red) metabolism: fructose-bisphosphate aldolase FBA class-I, *isiA*, Heme oxygenase, *idiA*, ferritin, *fur*, *isiB*, *pstS/sphX*, *pstAC*, *phoX*, *phnD*, *phnE*, *phnI*, *surE*, *ptxB*, *ptxC*; against measured dFe, DOP and DIP concentrations (nM). Pearson correlation coefficient *r* value indicated inside boxes (*n* = 17). Colour scale indicates significant positive correlation (violet), negative correlation (green) or non-significant (white) (Pearson parametric correlation test, *p* value < 0.05).

OGs known to respond to Fe or P availability (Supplementary Table 4) were further analysed to determine the in situ response of *Trichodesmium* populations to the varying nutrient availability across the transect. With the notable exception of flavodoxin (*isiB*) all Fe stress biomarkers measured were significantly correlated (Pearson test, *p* value < 0.05) to observed dFe concentrations as expected (Fig. 4). A negative correlation was seen for Fe-stress biomarkers including *idiA*/*futA*, chlorophyll-binding *isiA* [24, 84, 85], and heme oxygenase, responsible for heme degradation [25]. Further, upregulation of proteins lacking Fe cofactors was seen under the lowest Fe conditions for the non-metal binding fructose-bisphosphate aldolase class-I that may substitute the divalent metal binding fructose-bisphosphate aldolase class-II [21], but was not seen for *isiB* which can replace Fe-dependent ferredoxin in many cyanobacteria [26]. In addition, the Fe-storage protein ferritin [86] and ferric uptake regulators fur1/2 [87] were positively correlated with dFe, often upregulated under Fe-replete conditions. The availability of Fe thus impacted *Trichodesmium* physiology across the transect. However, transcription of Fe-stress related OGs even at the highest dFe concentrations suggests a response to Fe scarcity is common throughout the system relative to their needs, despite periodic dFe inputs in the NASG [19, 85].

Similar to Fe, all detectable OGs related to P stress, with the exception of one, were significantly negatively correlated with TDP (Pearson test, *p* value < 0.05) (Fig. 4). OGs for the high affinity phosphate-specific transporter *pst* were detected across samples. The inner membrane *pstA/C* OG was negatively correlated with DIP, whereas the phosphate-binding *pstS/sphX* OG (usually upregulated under low phosphate conditions), showed no statistical relation to the small observed gradient in DIP (Fig. 4) suggesting it is unresponsive to DIP [33] or that the low DIP concentrations across this transect did not vary enough to induce a significant transcriptional change. Transcripts for OGs involved in phosphite acquisition (PO_3_^3−^) *ptxB* and *ptxC* [36] were also observed to be negatively correlated to DIP. The DIP pool is typically dominated by phosphate rather than phosphite, and therefore we cannot determine any direct responses to phosphite concentration from these data.

Upregulation of OGs involved in DOP uptake can occur when DIP is low [33]. The rapid turnover time of *phoA* and *phoX* transcripts (coding for APs) makes them good P-availability biomarkers [22, 88]. Trace metal cofactors such as Fe and Zn likely play a role in AP activity, with the Fe-binding *phoX* potentially replaced by the Zn binding *phoA* when Fe becomes less available in the environment [72, 89]. In the present study, Fe-dependent *phoX* expression had a strong negative correlation with DOP (Pearson test, *p* value < 0.05) (Fig. 4) and positive correlation with the opposing gradient in dFe, while *phoA* expression was not detected. This contrasts with Rouco et al. [23], where *phoA* was detectable across basins, but with significantly lower transcription in the NASG relative to the low-Fe North Pacific Subtropical Gyre.

In addition, transcript abundances for OGs involved in acquisition of phosphonate *phnD,E,I*, were found to be significantly negatively correlated with DOP (Pearson test, *p* value < 0.05) (Fig. 4) [34]. The nucleotidase *surE* OG involved in P release from polyphosphate hydrolysis [90] was also strongly negatively correlated to DOP (*p* value < 0.05) (Fig. 4), potentially suggesting *Trichodesmium* was accessing P from intracellular polyphosphate molecules [22, 91]. Collectively these findings suggest that *Trichodesmium* is actively adapting to a dynamic nutrient system.

The inverse relationship between Fe and P encountered on this transect enabled direct evaluation of nutrient stress in *Trichodesmium* in situ (Fig. 4). Recognising that the observed surface nutrient concentrations (Fig. 1, Supplementary Fig. 3) represent the residuals remaining after biological removal [9, 14], collectively, the changes in expression of the suite of Fe and P regulated genes over the natural gradients in both Fe and P show that *Trichodesmium* populations shift their transcriptome in response to a dynamic nutrient regime. Moreover, for the mid-transect stations, relative upregulation of P stress markers compared to stations in the east, and Fe stress markers compared to stations in the west (Fig. 3), were suggestive of Fe-P co-stress.

### The N_2_ fixation transcriptome of *Trichodesmium*

**Fig. 5:**
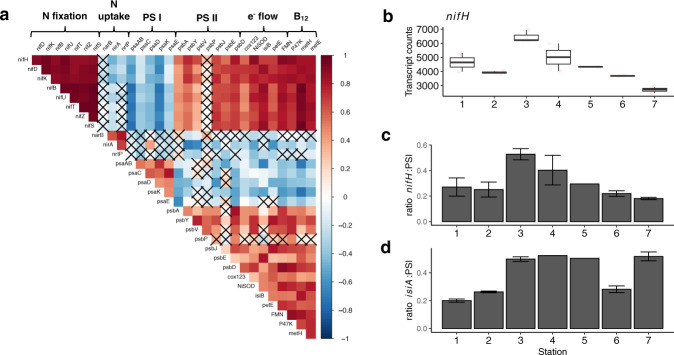
Co-regulation of nitrogen fixation metabolism. **a** Pearson correlation matrix for the normalised counts of the 17 samples for the nitrogenase genes against selected genes involved in fixed nitrogen uptake (nitrite/nitrate assimilation and transport), PSI, PSII, electron flow pathways, and vitamin B_12_ metabolism. Colour scale indicates *r* value with crosses on non-significant correlations (Pearson test, *p* value > 0.05). **b** Averaged DESEq2 normalised counts for *nifH* across stations. Ratios of the normalised counts per station for (**c**) *nifH*:PSI (p*saA*), and (**d**) *isiA:*PSI (*psaA*).

Increased expression of 11 nitrogenase OGs, including *nifHDKBUTZS* in the mid-transect (stations 3–5) occurred alongside other distinct transcription patterns (pattern II, Fig. 3b, Supplementary Fig. 4). The high rates of community N_2_ fixation and corresponding high *Trichodesmium* abundance in the mid-transect stations (Fig. 2b,c), alongside higher nitrogenase expression (Fig. 5) suggest enhanced *Trichodesmium* N_2_ fixation in this Fe-P co-stressed region [16, 17, 19]. Stations 3–5 had <0.77% variation in expression of OGs, indicating a consistent transcriptome expression pattern enabling this enhanced N_2_ fixation.

A range of other functional genes had expression patterns that were co-regulated with nitrogenase (Fig. 5). In addition to N_2_ fixation, Fe availability is key for photosynthesis and respiration electron transport metabolisms [92], since photosystems II and I, and nitrogenase require 3, 12 and 19 Fe atoms respectively [24]. *Trichodesmium* selectively sacrifices high Fe-requiring enzymes in a sequential manner under Fe scarcity [24]. Accordingly, the PSI core subunit OGs (*psaA/B)* were downregulated in the mid-transect stations, resulting in a *nifH*:PSI ratio for the mid-transect (0.41 ± 0.08) which were around double of those at either end (Fig. 5a, c), potentially reflecting allocation of Fe from PSI to nitrogenase in this region where N_2_ fixation rates were higher.

N_2_ fixation has a higher ATP:electron requirement (2:1) than C-fixation (1.5:1) and a component of PSI activity is normally employed in cyclic electron flow to generate the required extra ATP without generation of oxygen [30]. Reduced PSI transcript abundance relative to nitrogenase transcript abundance mid-transect (Fig. 5) might thus present an issue for ATP generation. A well-characterised compensation for reduction of PSI involves using the chlorophyll-binding protein *isiA* as an additional antenna for PSI [21, 84, 93]. *isiA* is regulated by the Fur operon with expression controlled by Fe availability [94] consistent with our observations (Fig. 4). Changes in *Trichodesmium isiA:*PSI ratios (Fig. 5d) reflect rates of N_2_ fixation (Fig. 2b) across the transect. Elevated *isiA:*PSI ratios (Fig. 5d) mid-transect where nitrogenase genes were upregulated thus potentially suggests one mechanism to increase the turnover rate of PSI to generate ATP for N_2_ fixation (Fig. 4; [21]). Further, the Fe-free electron carriers plastocyanin (*petE*) and flavodoxin (*isiB*), were also upregulated at the middle stations and thus positively correlated with the expression of nitrogenase OGs (Fig. 5a). As noted above, *isiB* was not correlated to Fe availability (Fig. 4); however, the correlation of Fe-free electron carriers with nitrogenase may suggest that the enhanced cellular requirements for Fe of nitrogenase, rather than simply dFe availability, regulate this response.

To enable N_2_-fixation, PSII activity may need to be reduced to prevent excess generation of O_2_ [30, 94] (Supplementary Fig. 7). However, PSII OGs were upregulated mid-transect (Fig. 5). While PSII activity can be regulated energetically [94], upregulation of PSII transcription alongside correlated upregulation of nitrogenase initially appears counterintuitive. However, diversion of electrons from water splitting to a variety of oxidases can represent an Fe-efficient mechanism for generating ATP from PSII activity without any net O_2_ production [95]. Interestingly, OGs encoding for three subunits of the cytochrome c oxidases and alternative respiratory oxidases (cox1-3) were upregulated at the mid-transect stations (Fig. 5a), potentially indicating such a mechanism. Similarly, nickel superoxide dismutase OG was significantly upregulated mid-transect (Fig. 5a), potentially indicating a method for scavenging oxygen radicals generated from PSII or alternative oxidases [96]. We thus suggest that under conditions of high cellular N_2_ fixation in a Fe-P co-stressed environment, *Trichodesmium* may employ a coordinated metabolic response relying on alternative electron flow strategies involving PSII water-water cycles and elevated *isiA*:PSI ratios to produce ATP [95] while allowing metalloenzyme reallocation strategies yielding Fe atoms from PSI.

Finally, several OGs related to cobalamin were present in the same gene expression profile II and correlated with nitrogenase *nifH* transcripts (Fig. 5a). This further supports the important role of *Trichodesmium* in the mid-transect marine microbiome, including *Trichodesmium* associated bacteria, as a source of both fixed N and essential vitamins [18, 43].

### Adaptation to a constructed Fe-P co-stressed niche

**Fig. 6:**
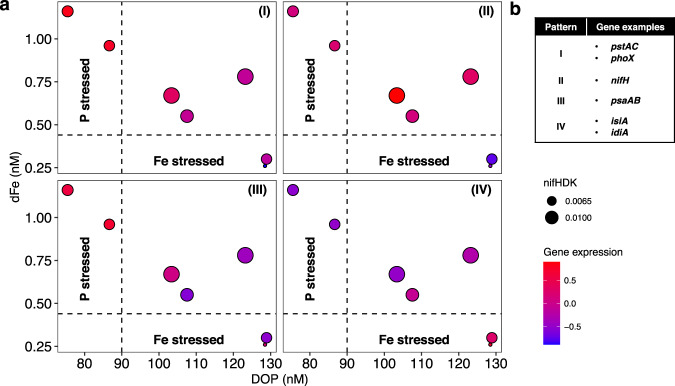
Transcription patterns related to resource availability in situ. **a** Transcription patterns (Fig. 3a) mapped into resource space. Dashed lines are ±2 standard deviations above the average nutrient concentration of dFe at stations 6–7 and DOP at stations 1–2. Colour scale indicates averaged normalised *z*-score across stations from the OGs for each of clusters I–IV (see Fig. 3). Symbol sizes indicate the averaged expression for the structural genes of nitrogenase *nifHDK*. **b** Gene examples found on each expression pattern plotted on (**a**).

The observed co-regulated expression patterns can be related to the well-established resource ratio theory [9, 14, 97]. The three broad environments along the longitudinal transect defined by Fe and P availability (Supplementary Fig. 3) were associated with the four statistically significant transcriptional profiles (Fig. 3b, Supplementary Fig. 4). Mapping the transcriptional patterns into ‘resource-space’ based on residual dFe and DOP availability enables definition of these four patterns of gene regulation along this transect. Expression up/downregulation was based on standard deviations variability of *z*-score scaled expression from each pattern. These are characterised by (I) upregulation when DOP < 90 nM; (II) upregulation when dFe > 0.44 nM and DOP > 90.4 nM; (III) downregulation when dFe > 0.44 nM and DOP > 90.4 nM; and (IV) upregulation when dFe < 0.44 nM (Fig. 6). With transcription profile (II) (Fig. 3b) associated with enhanced N_2_ fixation, which was characteristic of the mid-transect stations experiencing Fe and P co-stress [9, 19].

External forcing, including upwelling of waters with excess P (relative to N) and dust-borne Fe inputs, provides the drivers for biogeochemical gradients of the type sampled [13]. However, the activity of the organisms is also crucial in setting the state of the system, through depleting supplied nutrients to the point where they become limiting [11, 14]. Simple conceptual and numerical models would predict that abrupt shifts between single nutrient limitation patterns for both diazotrophs and non-diazotrophs should occur [9, 14, 97]. In contrast, the data presented here support previous experimental and physiological evidence of more widespread co-stress/limitation [19, 98]. Indeed, expression of both P and Fe stress biomarkers was observed and correlated with Fe and P availability across the whole transect (Figs. 3, 4). Although, as might be expected [14, 97], at the extremes of our transect, transcriptomic profiles indicated reduced nitrogenase expression associated with the highest Fe (east) or P (west) stress (Fig. 6a, b). The observations thus appear to suggest characterisation of the system in terms of large-scale gradients in the severity of co-stress.

Multiple eco-physiological mechanisms have been proposed for development of widespread regions of nutrient co-limitation [88, 99, 100]. Within the studied system we note that our observed transcriptomic patterns (Fig. 3b) suggest considerable metabolic flexibility linked to use of multiple chemical species of co-limiting nutrients, alongside compensation mechanisms which can alter the cellular requirements for these resources, both of which may contribute to enabling broad co-stressed conditions to persist [89]. Thus, in oligotrophic environments where *Trichodesmium* dominates the diazotrophic community, it may be responsible for the development and maintenance of a constructed Fe-P co-stressed niche (Fig. 6). Correspondingly, evolution might be expected to drive *Trichodesmium* towards having the highest growth rates [16, 17] and cellular nitrogenase activity [19] ultimately leading to highest fitness and ecological success under such conditions. However, the apparent lower cellular N_2_ fixation potential associated with increased nutrient stresses at either end of the transect suggest that any response to altered external forcing through changes in either P or Fe supply would still likely be a shift in the geographical locations of these niches [11], while enhanced input of either limiting resource would be expected to increase N_2_ fixation in a whole system sense.

### Summary

The natural inverse gradients in Fe and P availability captured in this study enabled a holistic view of the in situ molecular response of *Trichodesmium*. We report transcriptomic profiles (based upon multiple genes) suggesting that *Trichodesmium* responds to decreasing Fe or P availability by reducing N_2_ fixation capacity. *Trichodesmium* can maintain enhanced N_2_ fixation with a consistent and distinct metabolic profile at intermediate Fe-P concentrations, exhibiting a co-stressed expression profile coinciding with enhanced nitrogenase transcription over a large proportion of the North Atlantic gyre. Moreover, coordinated metabolic responses including Fe-reallocation, O_2_ reduction and a proposed alternative photosynthetic strategy to generate ATP without the requirement for PSI appear to be associated with enhanced nitrogenase expression in this region. Our results support previous suggestions that *Trichodesmium* has evolved to exploit conditions of Fe-P co-limitation [16, 17, 19], contributing to the global success of this keystone microbe. Co-limitation should thus be considered in ecological modelling of N_2_ fixation and understanding of system responses to altered external forcing.

## Supplementary information

Supplementary Information

Supplementary Table 4

Supplementary Table 5

## Data Availability

Data for this study has been deposited in NCBI and can be accessed as BioProject ID PRJNA721670 (metatranscriptome samples) and BioProject ID PRJNA721834 (Trichodesmium metagenome). The nutrient concentrations dataset has been submitted to the GEOTRACES Intermediate Data Product (IDP) 2021.
