# Supplementary material for: Transcriptional responses of Trichodesmium to natural inverse gradients of Fe and P availability
Source: ISME J. 2021 Nov 24;16(4):1055–64. doi: 10.1038/s41396-021-01151-1 (PMC8941076; doi:10.1038/s41396-021-01151-1)
Supplement: Supplementary file 1 — Supplementary Information [file 41396_2021_1151_MOESM1_ESM.docx]

**Supplementary information of**

**Transcriptional responses of *Trichodesmium* to natural inverse gradients of Fe and P availability**

Cerdan-Garcia, E.^1^; Baylay A.^1^; Polyviou D.^2^; Woodward E.M.S.^4^, Wrightson L.^3^, Mahaffey C.^3^, Lohan M.C.^1^; Moore C.M^1^; Bibby T.S.^1^; Robidart J.C.^2^

*Corresponding authors: [e.cerdangarcia@soton.ac.uk](mailto:e.cerdangarcia@soton.ac.uk); [j.robidart@noc.ac.uk](mailto:j.robidart@noc.ac.uk)

^1^Ocean and Earth Science, University of Southampton, UK SO14 3ZH,

^2^National Oceanography Centre, Southampton, UK SO14 3ZH,

^3^Earth, Ocean and Ecological Sciences, University of Liverpool, UK L69 3BX,

^4^Plymouth Marine Laboratory, UK PL1 3DH

**Supplementary Figure 1. Diazotroph *nifH* qPCR** *Trichodesmium* (grey) and UCYN-A1 (yellow) *nifH* gene abundance (gene copies L^-1^) quantified by qPCR across the 7 stations. Standard bars as the standard deviation of the technical and biological replicates.


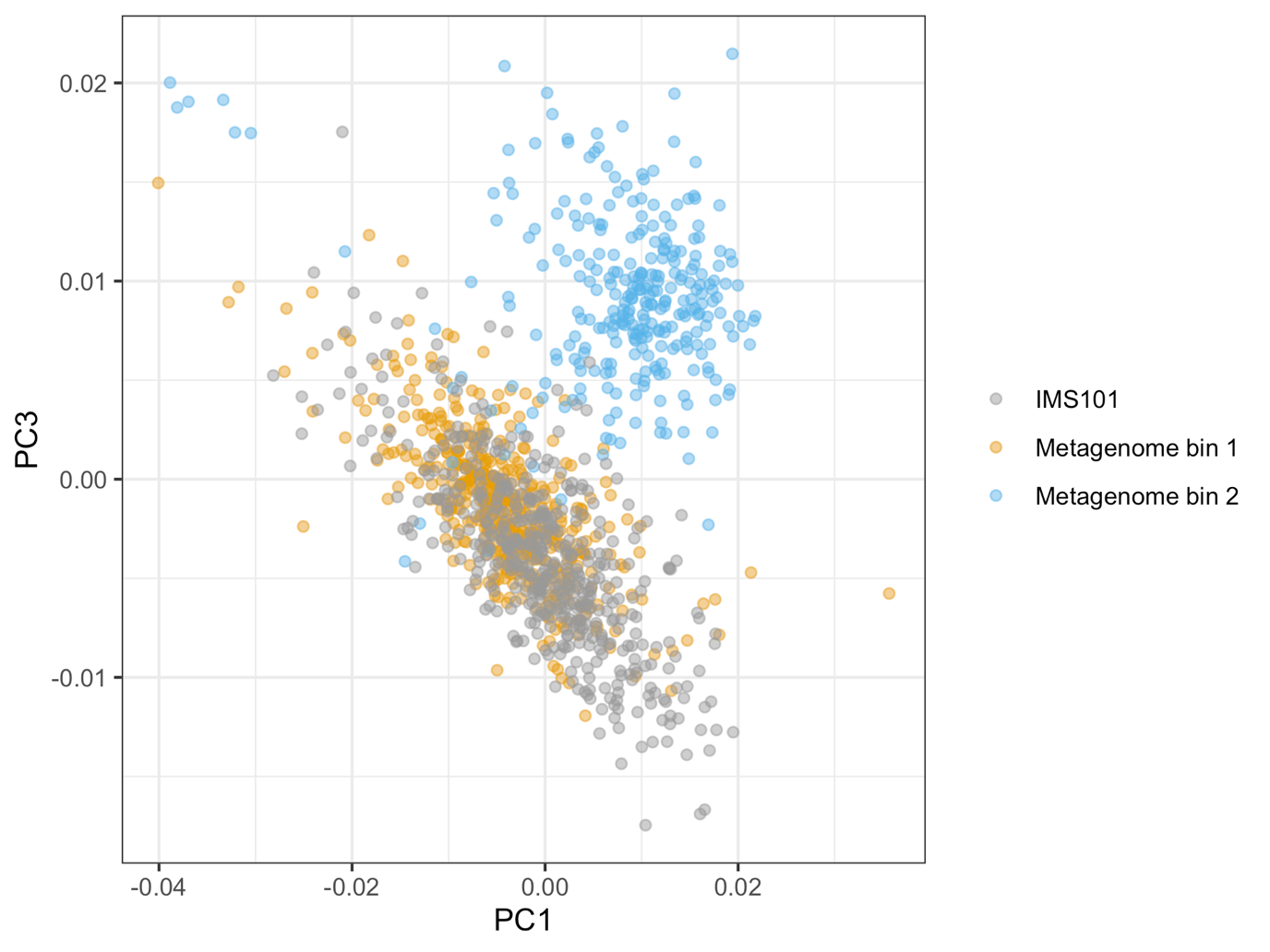
**Supplementary Figure 2. PCA ordination of metagenomic contigs based on tetranucleotide frequency profiles.** Tetranucleotide frequencies were computed from metagenome contigs > 5kb in length, as well as 500 randomly simulated fragments from *Trichodesmium erythraeum* IMS101 genome (mean length = 10kb, SD 1kb).

**Supplementary Figure 3.** (a) Relationship between dFe (nM) and TDP (nM) across the 7 sampling stations. Dashed lines are -2 standard deviations of the averaged nutrient concentrations for both dFe and TDP of the middle stations 3,4,5. (b) Ratio Iron to total P (dFe:TDP) from west (st 1,2) mid (3,4,5) and east (6,7), error bars show the standard deviation from the averaged values from each region.


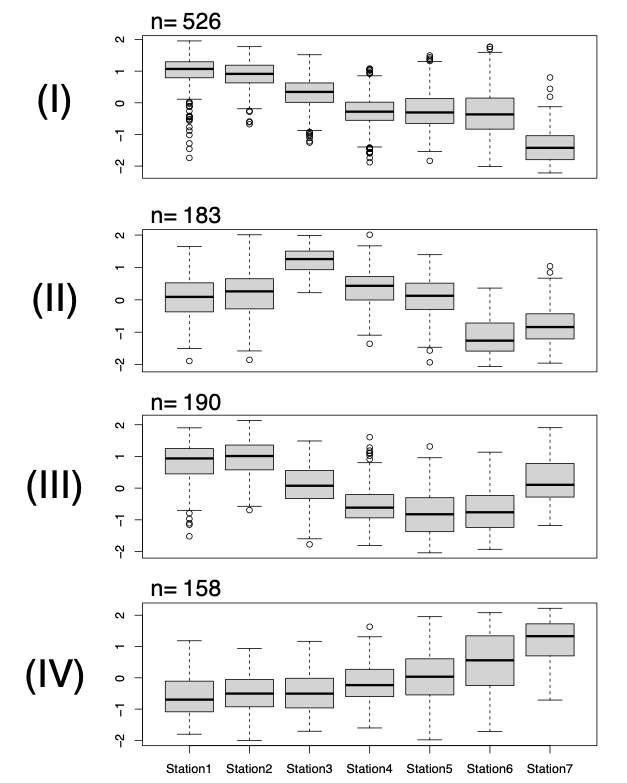


**Supplementary Figure 4. Heatmap patterns.** Transcription pattern averaged expression OG values across stations. Values are scaled to a z score (+/-2). Number of OGs from each profile pattern is noted on top of each plot.​


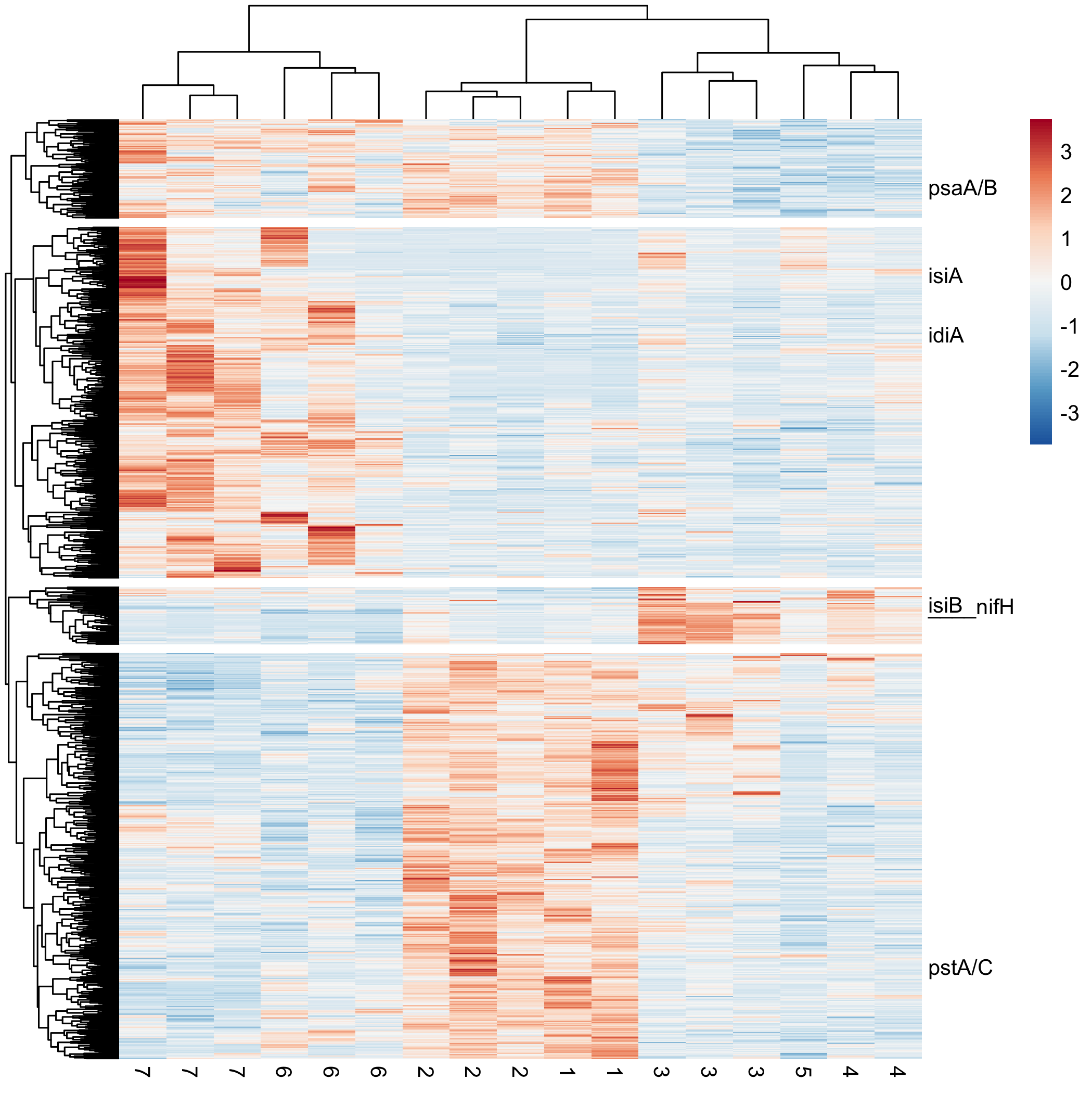


**Supplementary Figure 5. Transcript expression patterns of most Differentially Expressed Genes with metatranscriptome annotation against *Trichodesmium* metagenomic contigs from Rouco et al., 2018.** Following same approach as for Figure 3b, Columns are individual samples clustered based on basis of Euclidean distance annotated with station number. Colour scale indicates high (red) to low (blue) DESEq2 VST OG normalised gene abundance scaled per row. Four clusters of expression patterns were obtained from k-mean optimal clustering by Euclidean distance. These gene clusters as well as station clustering (station numbers at bottom) are the same as the analyses using metagenomic contigs from the current publication (Figure 3b). ​ Elected nutrient specific biomarkers are shown on the right.


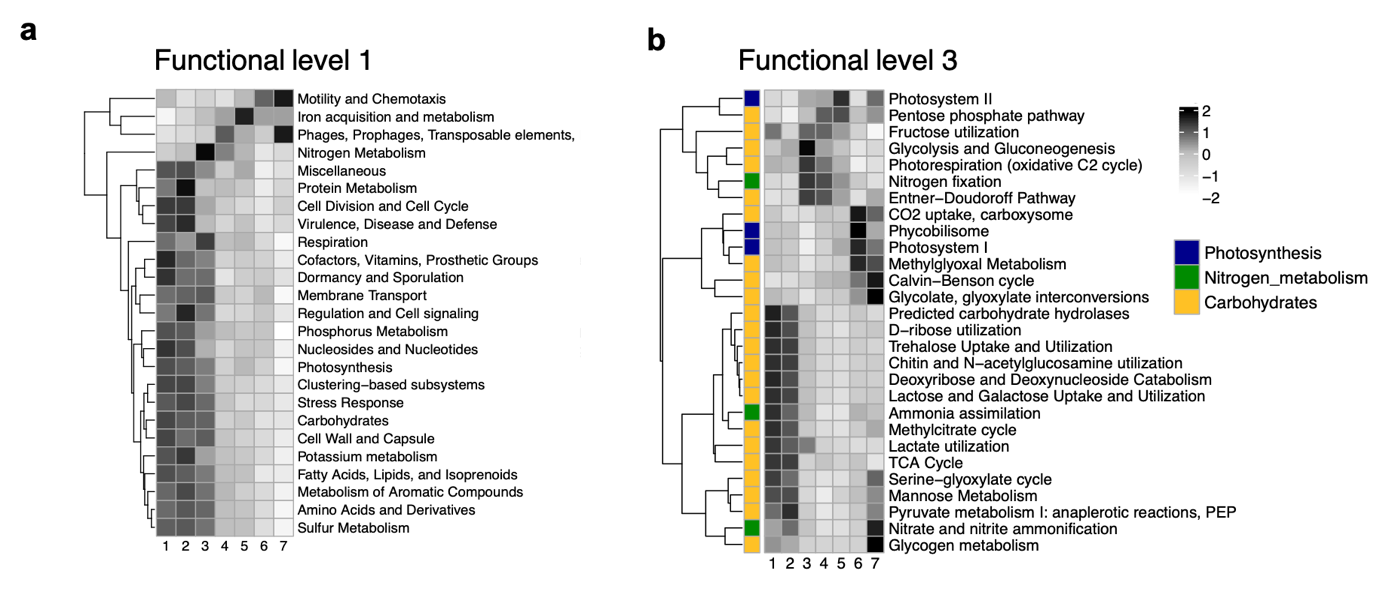


**Supplementary Figure 6. *Trichodesmium* functional analysis.** 1057 significantly differentially expressed OGs clustered into SEED subsystem categories levels 1 (a) and 3 (b) For Carbohydrate, Nitrogen metabolism and Photosynthesis. Stations (columns) are organised West-East. Category abundances coloured by sum of OG transcript abundance from more abundant (black) to lower (white) as scaled values per function/row (+/-2).

**
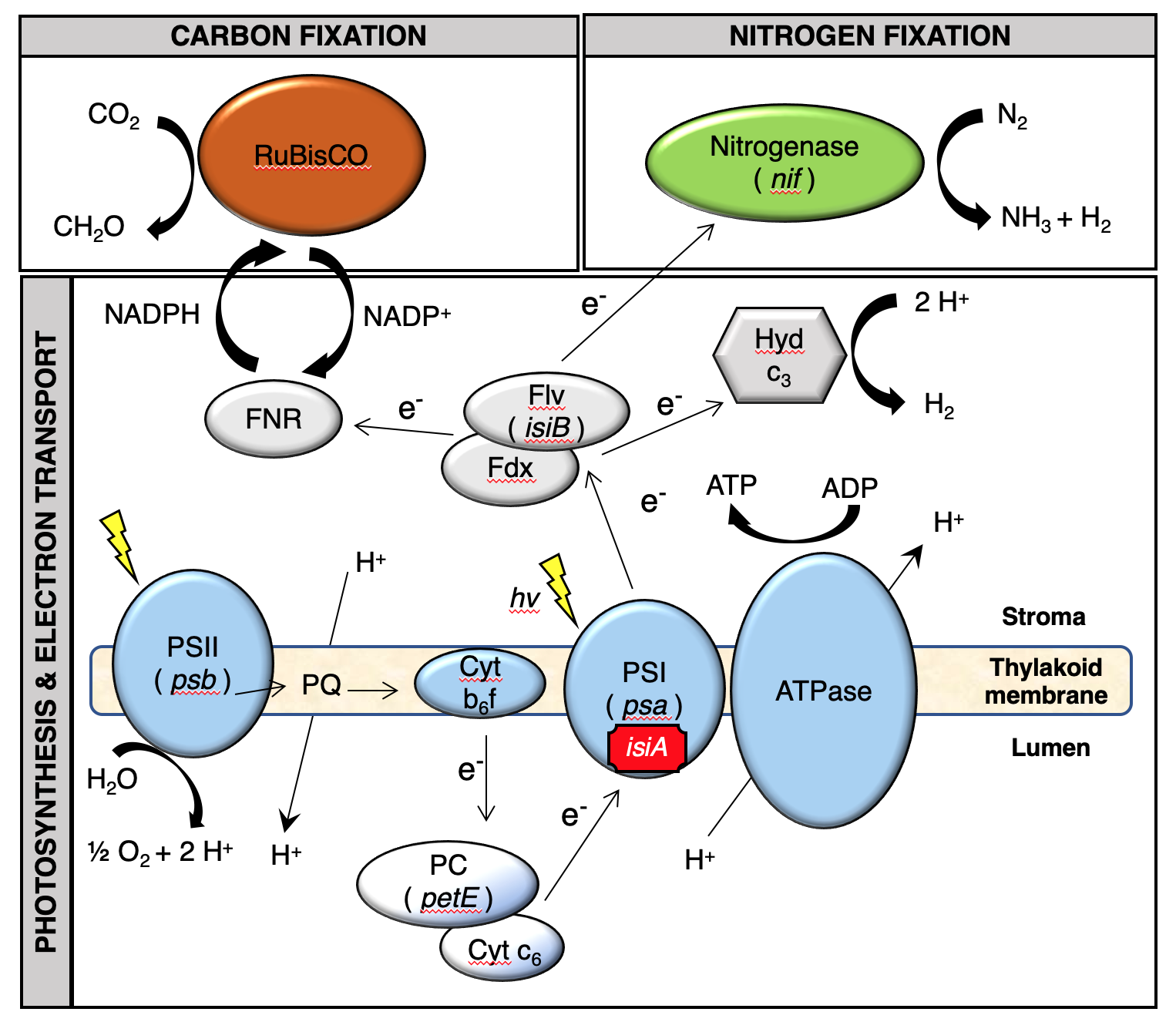
Supplementary Figure 7. Overview of the electron flow between the components of the photosynthesis, nitrogen fixation and carbon fixation in *Trichodesmium.*** This schematic representation includes the protein complexes and the genes encoding for these protein in italics between brackets. Abbreviations include: PSII - photosystem II, LHC - phycobilisome light harvesting complex, Cytb_6_f - cytochrome b_6_f complex; PSI - photosystem I; ATPase - ATP synthase; Pc – plastocyanin; Cyt c_6_ - cytochrome c_6_; PQ – plastoquinone; Fd – ferredoxin; FNR - ferredoxin:NADP^+^ reductase; I*siA* - iron stress induced protein; ATP - adenosinetriphosphate; ADP - adenosine diphosphate; NADP^+^/H – Nicotinamide adenine dinucleotide phosphate; RuBisCO - Ribulose-1,5- bisphosphate carboxylase/ oxygenase. Also shown in this figure the chlorophyll-binding protein *isiA* which associates with PSI under Fe stress (Bibby et al., 2001). Figure adapted from Snow et al., (2015).

| **Region** | **Station** | **cleaned reads** | **mRNA** | **Mapped to *Trichodesmium* IMS 101** | | **Mapped to Metagenome** | | | | | | **OGs present per station** | **Unique OGs of each station** | **OGs present per region** |
| --- | --- | --- | --- | --- | --- | --- | --- | --- | --- | --- | --- | --- | --- | --- |
|  |  |  |  | **SAMSA2 RefSeq NCBI** | **%** | **Mapped to CDS** | **Mapped to non-coding region** | **Unmapped** | **% Mapped to CDS** | **% Mapped to non-coding region** | **% Unmapped** |  |  |  |
| W | 1 | 1703663 | 1668176 | 597377 | 35.06% | 772454 | 363985 | 554649 | 45.68% | 21.52% | 32.80% | 3829 | 0 | 3940 |
| W | 1 | 1739872 | 1310271 | 429797 | 24.70% | 508634 | 244943 | 559510 | 38.74% | 18.65% | 42.61% |  |  |  |
| W | 2 | 944381 | 786124 | 240437 | 25.46% | 363140 | 190575 | 246116 | 45.40% | 23.83% | 30.77% | 3868 | 0 |  |
| W | 2 | 1003627 | 889872 | 290408 | 28.94% | 425707 | 212687 | 266571 | 47.04% | 23.50% | 29.46% |  |  |  |
| W | 2 | 2541987 | 2260415 | 767356 | 30.19% | 1108670 | 473169 | 713283 | 48.31% | 20.62% | 31.08% |  |  |  |
| M | 3 | 1410723 | 1301296 | 442069 | 31.34% | 561429 | 273198 | 483012 | 42.61% | 20.73% | 36.66% | 3910 | 0 | 3985 |
| M | 3 | 1665071 | 1486555 | 494608 | 29.70% | 650894 | 250223 | 603821 | 43.25% | 16.63% | 40.12% |  |  |  |
| M | 3 | 1168287 | 1105999 | 418563 | 35.83% | 422842 | 189615 | 506113 | 37.80% | 16.95% | 45.25% |  |  |  |
| M | 4 | 1589320 | 1382945 | 472143 | 29.71% | 590518 | 309057 | 501471 | 42.15% | 22.06% | 35.79% | 3892 | 0 |  |
| M | 4 | 1785685 | 1712377 | 542770 | 30.40% | 767685 | 373476 | 598067 | 44.14% | 21.47% | 34.39% |  |  |  |
| M | 5 | 635922 | 619457 | 224193 | 35.25% | 276265 | 130435 | 220457 | 44.05% | 20.80% | 35.15% | 3515 | 1 |  |
| E | 6 | 1328092 | 1159455 | 316628 | 23.84% | 400201 | 290054 | 482372 | 34.13% | 24.74% | 41.14% | 3838 | 0 | 3948 |
| E | 6 | 1439789 | 1368663 | 461953 | 32.08% | 593407 | 319964 | 471879 | 42.84% | 23.10% | 34.06% |  |  |  |
| E | 6 | 916586 | 856828 | 234027 | 25.53% | 324283 | 229215 | 312555 | 37.44% | 26.47% | 36.09% |  |  |  |
| E | 7 | 490555 | 471088 | 169803 | 34.61% | 174431 | 90007 | 211616 | 36.64% | 18.91% | 44.45% | 3829 | 1 |  |
| E | 7 | 1102990 | 1056761 | 383380 | 34.76% | 448404 | 201501 | 418264 | 41.98% | 18.86% | 39.16% |  |  |  |
| E | 7 | 1250112 | 1205425 | 446399 | 35.71% | 490787 | 210531 | 513784 | 40.39% | 17.33% | 42.28% |  |  |  |

**Supplementary Table 1: Read number and mapping percentages for the data samples**. These include (A) cleaned and mRNA counts, (B) Number of mRNA reads and percentage mapped to *Trichodesmium* RefSeq IMS101: ; (C) Number of reads and percentages mapped to the custom *Trichodesmium* metagenome (CDS, non-coding region and unmapped), (D) Number OGs present ( >1 read) per station and region, and OGs unique to each station/region (> 1 read and absent on the rest) of the total 4044 OGs.

**Supplementary Table 2.** Measured surface concentrations for organic phosphorus compounds for the sampling stations across the transect.

| **Station** | **Longitude** | **MLD** | **TDP ML conc** | **DOP** | **SRP** | **N+N** | **NH4** |
| --- | --- | --- | --- | --- | --- | --- | --- |
|  | **W** | **m** | **nmol L^-1^** | **nmol ^L-1^** | **nmol L-1** | **nmol L-1** | **nmol L-1** |
| **2** | 54 | 180.4 | 67.0 | 58.6 | 6.8 | 4.8 | 23.8 |
| **3** | 50 | 199.0 | 79.0 | 77.3 | 6.7 | 15.2 | 35.4 |
| **4** | 44.95 | 160.6 | 115.0 | 106.5 | 8.0 | 2.5 | 4.8 |
| **5** | 40 | 140.5 | 110.1 | 103.6 | 5.2 | 12.8 | 12.8 |
| **6** | 35.87 | 120.2 | 132.7 | 127.5 | 5.3 | 14.2 | 8.4 |
| **7** | 31 | 100.4 | 154.1 | 151.4 | 7.0 | 6.3 | 8.4 |

**Supplementary Table 3.** Differentially expressed OGs (DEOGs) between individual stations in number of OGs (top right) and percentage of the 4044 total OGs (bottom left). Colouring on grey scale from lower (lighter colour) to higher similarity values (darker).

| **Station** | **1** | **2** | **3** | **4** | **5** | **6** | **7** |
| --- | --- | --- | --- | --- | --- | --- | --- |
| 1 |  | 3 | 175 | 339 | 57 | 572 | 870 |
| 2 | 0.07% |  | 204 | 316 | 44 | 685 | 935 |
| 3 | 4.33% | 5.04% |  | 31 | 11 | 401 | 672 |
| 4 | 8.38% | 7.81% | 0.77% |  | 0 | 142 | 396 |
| 5 | 1.41% | 1.09% | 0.27% | 0% |  | 49 | 165 |
| 6 | 14.14% | 16.94% | 9.92% | 3.51% | 1.12% |  | 358 |
| 7 | 21.51% | 23.12% | 16.62% | 9.80% | 4.08% | 8.85% |  |

**Supplementary methods.**

**Nitrogen fixation methods.**

Samples were incubated for 24 hours (pre-dawn to pre-dawn) in a temperature-controlled container set at the local mean mixed layer depth temperature (25 to 27°C). Incubations followed a 12:12 h light cycle using daylight LED light panels (Part no: LED-PANEL-300-1200-DW and LED-PANEL-200-6-DW, Daylight White, supplier Power Pax UK Limited). The solubility of N_2_ was determined following methods by Hamme and Emerson, 2004. The ratio between N_2_ mole masses 28, 29 and 30 relative to argon, was measured by record volts for 28, 29 and 30 for individual samples using a Hiden membrane-inlet mass spectrometer. The bubble was removed at the same time as a subsample of seawater, which was analysed on board.

Agitated bottles achieved an atom% enrichment of 10 ± 2 (n=252). Atom %15N enrichment was calculated using a spreadsheet created by John Dore for N_2_ fixation measurements at the University of Hawaii. Comparison with equation 4 in White et al 2020 (page 133) reveal that the resulting atom% enrichments are the same. The minimum detectable rates were calculated as the min change in APN (assumed to be 0,00146%) x PN concentration (µM) x N_2_% enrichment ^-1^. The limit of detection was 0.07 nmol L-1 (0.0470.141, n=45). Mean PN mass on filters (± standard deviation) was 27 ± 6 µg N (n=244), equating to a mean ± standard deviation concentration of 0.45 ± 0.18 µM (n=244). The T0 was based on the mean of an AMT data set for the subtropical Atlantic (1.92± 0.17‰, Mahaffey, 2004).

**Quantitative PCR, primers, reagents, concentrations and conditions**

**qPCR for *nifH*:** Reactions were run in triplicate for each sample. For *Trichodesmium*, 30 µl reaction mixes contained 1 x Accuprime PCR Master Mix (Invitrogen, CA, USA), 2.5 mM MgCl2, 0.2 µm FAM-labelled probe’ and 0.4 µM *Trichodesmium* *nifH* F primer and *Trichodesmium* *nifH* R primer as described (Church et al., 2005; Robidart et al., 2014). For UCYN-A, the same reaction conditions above were used but with primers and probe targeting the UCYN-A1 *nifH* sequence: UCYN-A *nifH* F, UCYN-A *nifH* R, UCYN-A nifH FAM-labelled probe (Church et al., 2005). Negative controls showed no amplification, and the efficiency of the qPCR reactions ranged from 98.5% to 103.1%, with an average of 101.3% for *Trichodesmium*; and from 98.6% to 107.2%, with an average of 103.6% for UCYN-A.

|  | ***nifH* primers and probes (5’-3’)** | |
| --- | --- | --- |
| ***Trichodesmium***  (Church et al., 2005; Robidart et al., 2014) | **Forward** | GACGAAGTATTGAAGCCAGGTTTC |
|  | **Reverse** | CGGCCAGCGCAACCTA |
|  | **Probe** | CATTAAGTGTGTTGAATCTGGTGGTCCTGAGC |
| **UCYN-A**  (Church et al., 2005) | **Forward** | AGCTATAACAACGTTTTATGCGTTGA |
|  | **Reverse** | ACCACGACCAGCACATCCA |
|  | **Probe** | TCTGGTGGTCCTGAGCCTGGA |

**References from supplementary information**

1. Church MJ, Jenkins BD, Karl DM, Zehr JP. Vertical distributions of nitrogen-fixing phylotypes at Stn ALOHA in the oligotrophic North Pacific Ocean. Aquatic Microbial Ecology. 2005;38(1):3-14.
2. Hamme, R. C., & Emerson, S. R. (2004). The solubility of neon, nitrogen and argon in distilled water and seawater. *Deep Sea Research Part I: Oceanographic Research Papers*, *51*(11), 1517-1528.
3. Mahaffey, C., Williams, R. G., Wolff, G. A., & Anderson, W. T. (2004). Physical supply of nitrogen to phytoplankton in the Atlantic Ocean. *Global Biogeochemical Cycles*, *18*(1).
4. Robidart, J.C., Church, M.J., Ryan, J.P., Ascani, F., Wilson, S.T., Bombar, D., Marin, R., Richards, K.J., Karl, D.M., Scholin, C.A. and Zehr, J.P. (2014). Ecogenomic sensor reveals controls on N 2-fixing microorganisms in the North Pacific Ocean. *The ISME journal*, *8*(6), 1175-1185.
5. White, A. E., Granger, J., Selden, C., Gradoville, M. R., Potts, L., Bourbonnais, A., ... & Chang, B. X. (2020). A critical review of the 15N2 tracer method to measure diazotrophic production in pelagic ecosystems. *Limnology and Oceanography: Methods*, *18*(4), 129-147.
